# A comprehensive accident investigation system for assisted driving vehicles: Addressing complexities in fault determination and responsibility allocation

**DOI:** 10.1371/journal.pone.0328156

**Published:** 2025-07-14

**Authors:** Yanbin Hu, Wenhui Zhou

**Affiliations:** Research Institute for Road Safety of the Ministry of Public Security, Beijing, China; Beijing Institute of Technology, CHINA

## Abstract

China’s autonomous vehicle accidents, fault determination has grown increasingly complex due to the undefined legal status of autonomous driving systems. To assign responsibility accurately, it is crucial to consider the system’s autonomy and decision-making capabilities. Furthermore, the lack of specialized technical equipment and software for data collection, analysis, and processing hinders rapid and precise problem identification, impacting accident handling efficiency and accuracy. To address this, a tailored comprehensive accident investigation system for assisted driving vehicles has been developed. This system comprises subsystems for traffic accident boundary analysis, control action safety analysis, causal scene restoration, and investigation report output. It systematically extracts accident evidence, analyzes causes, traces hidden hazards, and determines responsibility. By integrating these subsystems, the system offers a structured and comprehensive framework for intelligent vehicle accident investigations, enhancing overall efficiency and clarity.

## 1 Introduction

In recent years, China’s intelligent driving vehicle industry has developed rapidly, especially the number of cars with assisted driving function has increased rapidly. At the same time, traffic accidents involving assisted driving vehicles show a tendency of multiple, and safety problems are prominent [1]. In the process of traffic accident investigation involving assisted driving vehicles, due to the intervention of assisted driving function [2]. As a result, accidents under the strong coupling of multiple factors, such as “human-vehicle-road-environment", are difficult to obtain key evidence, difficult to analyze the process, difficult to trace hidden dangers, difficult to determine responsibility [3], and other problems, which affect the accuracy, science and fairness of the identification of traffic accident responsibility [4]. Kim Heesoo [5] collected reports used in existing traffic accident investigations, autonomous driving-related reports and literature, and accident videos involving autonomous driving to build investigation items [14], reviewed the items required for investigation in the event of a conventional vehicle accident and added additional investigation items deemed necessary to be reviewed in addition to the existing reports [6]. Evtiukov Sergei [7] identify the most relevant parameters of the vehicle condition and the road environment necessary for automobile technical expert evaluation (e.g. the friction coefficient [15], vehicle braking performance under different loads on all categories of roads with different types of road surface, roughness, wheel tracking, hydraulic roughness) and to obtain their actual values [16]. Hu Lin [8] based on the China In-Depth Accident Study database, taking the number of casualties in the accident as evaluation indicators, the grey cluster analysis was used to classify the drivers into four accident risk rank. Dennis Buddy Saputra [9] reviews various methods reconstructing an accident scene, such as photogrammetry and laser scanning, as well as elaborating the relevant data that has already available in the vehicle and from the infrastructure [10]. The current investigation method can not determine the cause of traffic accidents according to the output index data [11], and the current traffic accident analysis system can not accurately identify the cause of traffic accidents [12], especially for vehicles with assisted driving function [13]. Recent advancements in accident investigation methodologies have increasingly focused on integrating artificial intelligence (AI) and real-time data analytics to overcome the limitations of traditional approaches [21]. Studies have highlighted the need for dynamic evidence collection frameworks that adapt to accident complexity. For instance, AI-driven platforms now leverage vehicle black box data, road infrastructure sensors [23], and environmental APIs to reconstruct accidents with temporal precision, prioritizing critical evidence through machine learning-based triage systems [25]. These frameworks address longstanding gaps in manual investigation workflows [26], particularly in scenarios involving rapid system failures or multi-entity interactions [27]. Concurrently, interdisciplinary efforts have explored the legal and ethical dimensions of liability determination in assisted driving contexts [28]. Scholarly work has emphasized the necessity of tiered responsibility models that disentangle contributions from system malfunctions, human errors [31], and external environmental factors [29]. Digital twin technologies have emerged as pivotal tools in this domain [32], enabling investigators to simulate counterfactual accident scenarios and quantify the causal impact of human-machine interaction dynamics [30]. While these innovations enhance analytical rigor, challenges persist in harmonizing technical precision with jurisdictional legal standards [22], necessitating further research into cross-domain frameworks that balance algorithmic accountability with procedural fairnes [24]. Investigating accidents involving assisted driving vehicles presents several challenges. First, systematically extracting accident evidence is crucia [17]. Next, accurately analyzing the causes of these accidents is essential. Tracing potential hazards effectively is another hurdle. Lastly, determining accident responsibility is vital [18]. In the complex and rapidly evolving world of assisted driving vehicles [19], ensuring the accuracy and reliability of the accident investigation system faces numerous technical and practical challenges [20]. Our system addresses these issues by integrating multiple subsystems. These include traffic accident boundary analysis, control action safety analysis, causal scene reconstruction, and investigation report output. This comprehensive and systematic approach enhances the efficiency of accident investigations. Furthermore, it provides a scientific foundation for accident prevention and responsibility determination.

To tackle the intricate challenges of investigating and assigning liability in accidents involving assisted driving vehicles, particularly those where the interplay of assisted driving functions, amidst the highly interconnected dynamics of the driver, vehicle, road, and environment, has resulted in collisions, we offer comprehensive methodological support across various stages. This includes evidence acquisition, process analysis, hazard tracing, and responsibility determination. Our aim is to scientifically and rationally pinpoint the crucial factors influencing accidents involving assisted driving vehicles, while also providing a technical roadmap for hazard tracing. This ensures the precision, scientific rigor, and impartiality of liability determination in such accidents. To fulfill this research objective, the comprehensive factor investigation system for assisted driving vehicle accidents must encompass, but is not restricted to, functions like accident boundary delineation, control processing, safety evaluation of control actions, on-site reconstruction of accident causes, and the generation of investigation reports. A comprehensive factors investigation system for traffic accidents involving assisted driving vehicles is introduced, encompassing subsystems for traffic accident boundary analysis, control processing, control action safety analysis, traffic accident causal scene recovery, and investigation report export. The traffic accident boundary analysis subsystem defines the content boundary for traffic data necessary to analyze accident causes and collects relevant data accordingly. The control action safety analysis subsystem establishes a safety analysis model for control actions, enabling the identification of unsafe factors in traffic accidents. The traffic accident causal scene recovery subsystem utilizes the unsafe factors identified by the control action safety analysis subsystem to restore the accident scene and obtain detailed information about the auxiliary driving vehicle involved. Finally, the investigation report export subsystem generates an in-depth investigation report based on the causal scene information of the intelligent vehicle. This system ensures clarity, accuracy, and efficiency in the investigation of traffic accidents involving assisted driving vehicles. In Table 1 provides a detailed list of the current methods for investigating traffic accidents involving assisted driving vehicles, including the method names, main characteristics, advantages, limitations, and comparisons with the method proposed by our system.

**Table 1:** Comparison of accident investigation methods for assisted driving vehicles.

Method	Advantages	Limitations	Control Action Safety Analysis Model
Traditional Accident Reconstruction	Mature methodology with broad applicability across accident scenarios	Limited capability in analyzing complex system interactions	Provides technical failure analysis framework complementary to traditional methods
Data-Driven Analysis	Objective data processing with quantifiable results	Reliance on complete data recording systems	Integrates data-driven approach with layered failure identification
Simulation Replication	Cost-effective repeatable testing under controlled conditions	Accuracy limitations from modeling simplifications	Offers scenario classification system for simulation design guidance
Expert Evaluation	Holistic perspective incorporating legal/technical/human factors	Subjectivity in expert judgment	Provides structured framework to enhance evaluation objectivity
AI-Assisted Analysis	Efficient processing of multi-source heterogeneous data	Black-box issue affecting interpretability	Introduces explainable AI framework for improved analysis
Control Action Safety Analysis Model	Systematic failure mode identification with precise scenario coverage	High implementation cost requiring interdisciplinary collaboration	N/A

## 2 System framework design

**Fig 1:**
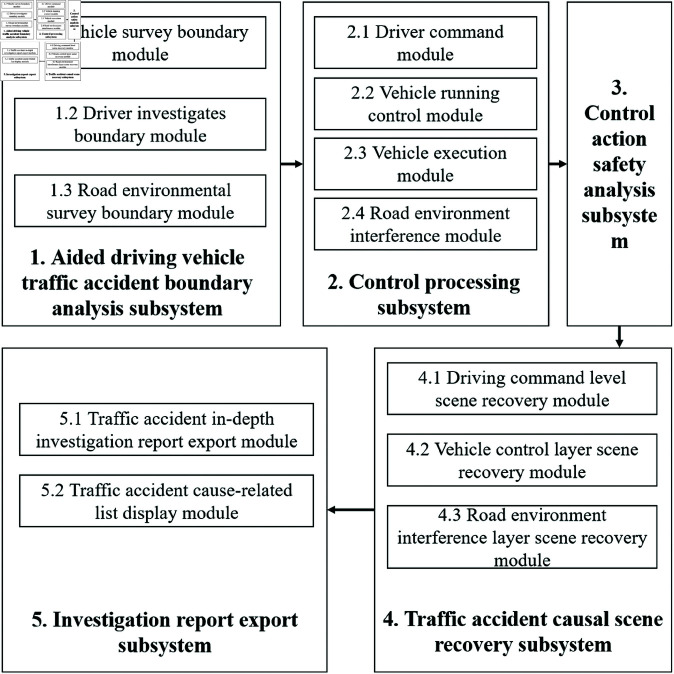
Framework of comprehensive factors investigation system for traffic accidents of assisted driving vehicles.

The Systems-Theoretic Accident Model and Processes (STAMP), widely applied in the field of safety production hazard identification, provides a theoretical foundation and framework for system safety analysis, emphasizing the development of accident models and the revelation of root causes. Based on the STAMP model’s principles, this article analyzes the overall function, processes, and control strategies involved in the occurrence of assisted driving vehicle accidents, identifying potential poor control actions or failure mechanisms that could lead to traffic accidents. This leads to the refinement of the System-Theoretic Process Analysis (STPA), a method specifically implemented for safety analysis under the guidance of STAMP. STPA and STAMP support and complement each other, jointly providing a comprehensive and effective method and tool for system safety analysis. The specific implementation focuses on investigation items and methods related to vehicles, drivers, roads, and environmental factors in assisted driving vehicle traffic accidents. In Fig 1, the comprehensive factors investigation system for assisted driving vehicle traffic accidents clearly shows the communication links between subsystems. The traffic accident boundary analysis subsystem defines the scope of traffic data needed for accident analysis. It connects with the control processing subsystem, which establishes the relationships between various traffic data. The control action safety analysis subsystem, in turn, defines a safety model for control actions.

By using this safety model and the relationships from the control processing subsystem, unsafe factors in accidents are identified. These factors help determine the cause of the accidents. The causal scene recovery subsystem, linked to the control action safety analysis subsystem, reconstructs the accident scene based on the unsafe factors.

Once the causal scene information is obtained, the investigation report export subsystem generates a detailed report. It uses the information from the causal scene recovery subsystem about the assisted driving vehicles involved.

The traffic accident boundary analysis subsystem has three modules. The vehicle investigation boundary module sets the scope for vehicle data in accidents involving assisted driving. The driver investigation boundary module defines the scope for driver data. And the road environment investigation boundary module establishes the scope for environmental data.

The control processing subsystem includes several modules. The driving command module interacts with the vehicle and driver investigation boundary modules. The vehicle running control module and vehicle execution module work with the vehicle investigation boundary module. The road environment interference module connects with the road environment investigation boundary module.

The driving command module has two submodules: auxiliary driving and human driving. The human driving submodule communicates directly with the vehicle running control module and also through the auxiliary driving submodule. The road environment interference module provides data that affects both the vehicle running control module and the driving command module.

The driving command module sends driving commands based on input from the vehicle and driver investigation boundary modules. The vehicle running control module receives these commands and sends control states to the driving command module and the vehicle execution module. The vehicle execution module, as the actuator, responds by executing vehicle actions.

The traffic accident causal scene recovery subsystem has modules for the driving command layer, vehicle control layer, and road environment interference layer. These modules interact with the control action safety analysis subsystem. The road environment interference layer module uses cameras and simulation software to trace, measure, and analyze the road environment, reconstructing the accident scene.

The vehicle control layer module reconstructs vehicle data using onboard electronics, including movement, collisions, angles, speeds, and acceleration/deceleration rates. The driving command layer module restores the operation sequences of both the auxiliary driving system and human driver using onboard electronics and video data.

The investigation report export subsystem has two modules. The traffic accident in-depth investigation report export module generates textual reports on unsafe factors in accidents, based on the recovered scenes. The traffic accident cause-related list display module exports tables of these unsafe factors, also derived from the recovered accident scenes.

## 3 How the system works

The traffic accident boundary analysis subsystem within the system serves to delineate the content boundaries of traffic data essential for analyzing the causes of traffic accidents. It subsequently collects traffic data in accordance with these defined content boundaries. The term “content boundary" encompasses specific data items, value ranges, and other constraints pertinent to the investigation of traffic accidents involving assisted driving vehicles. This includes, but is not limited to, the configuration information and functionality of the assisted driving system, its dynamic operations, and vehicle braking maneuvers.

The control processing subsystem is tasked with establishing the logical relationships among various types of traffic data. Specifically, it categorizes the traffic data gathered by the traffic accident boundary analysis subsystem for assisted driving vehicles into data modules related to human driving operations, auxiliary driving operations, and vehicle control actions. This categorization clarifies the data flow relationships among each module, thereby determining the logical interplay between diverse traffic data types. For instance, data pertaining to human driving operations is first directed to the auxiliary driving module for command generation, which is then relayed to the vehicle control action module for actual steering wheel and brake control signal execution. The control processing subsystem elucidates the data flow dynamics within the vehicle during operation and how these data interact to facilitate vehicle function.

Subsequently, the control action safety analysis subsystem defines the control action safety analysis model, which is utilized to conduct corresponding calculations based on the data flow logical relationships established by the control processing subsystem. This analysis qualitatively and quantitatively identifies unsafe human control actions, unsafe auxiliary driving control actions, and unsafe environmental factors within the traffic data. By summarizing these unsafe factors, the subsystem ultimately determines the underlying causes of traffic accidents.

Drawing upon the unsafe factors identified by the control action safety analysis subsystem, the traffic accident causal scene recovery subsystem is employed to reconstruct the traffic accident scene. The reconstructed information can be presented in formats such as animation or text description, thereby enhancing users’ ability to ascertain the accident cause and fostering a more intuitive understanding. Furthermore, the investigation report export subsystem generates an analytical report on the reconstructed traffic accidents, directly pinpointing the core, key causes, and the impact of each cause. This report facilitates user and law enforcement comprehension of the original causes of traffic accidents and offers actionable insights. Additionally, the report’s analysis provides reasonable suggestions, reminding users and law enforcement to enhance vehicle driving assistance functions, improve road conditions, and thereby reduce the likelihood of future traffic accidents, ultimately enhancing traffic safety.

### 3.1 Accident data collection

The Vehicle Investigation Boundary Module within the Traffic Accident Boundary Analysis Subsystem serves to delineate the initial content boundary pertinent to assisted driving in traffic accidents and subsequently gather vehicle data based on this defined boundary. Specifically, this module is primarily tasked with collecting vehicle-related investigative data in accidents involving assisted driving vehicles, while also establishing the content boundary for such investigative data. Illustratively, the content boundary encompasses configuration information and functionalities of the assisted driving system, its dynamic operational capabilities, and the braking mechanisms of the vehicle.

Consequently, the data collected may encompass the control actions executed by the auxiliary driving system on the vehicle’s driving control module, encompassing inputs such as braking, throttle modulation, steering adjustments, headlight activation, angle adjustments, turn signal activation, and other relevant operations. Additionally, it may also involve the control actions initiated by the vehicle’s driving control module towards the auxiliary driving system, including inputs related to road information, targets, vehicle power, signal lights, traffic signs, vehicle positioning, control status, throttle state, braking status, and more.

### 3.2 Data analysis and model construction

The Driving Command Module of the Control Processing Subsystem interfaces with both the Vehicle Investigation Boundary Module and the Driver Investigation Boundary Module. The Vehicle Running Control Module and the Vehicle Execution Module are interconnected with the Vehicle Investigation Boundary Module, while the Road Environment Interference Module communicates with the Road Environment Investigation Boundary Module.

The Control Processing Subsystem is designed to input end-cloud data, collected by the Traffic Accident Boundary Analysis Subsystem for assisted driving vehicles, into the Driving Command Module, Vehicle Running Control Module, (note: Vehicle Running Control Module is mentioned twice consecutively in the original text, possibly a repetition; assuming the second mention should be omitted or corrected if intended for another module), and Road Environment Interference Module through a dedicated data access port. The Driving Command Module of the Control Processing Subsystem is linked to the Vehicle Driving Control Module via either the Auxiliary Driving System or the human driver.

In application, the Driving Command Module issues corresponding driving commands based on data from the Vehicle Investigation Boundary Module and the Driver Investigation Boundary Module. Its input is facilitated by the controller and other vehicle information inputs. The Vehicle Travel Control Module, connected to the Vehicle Execution Module, receives these driving commands and outputs the corresponding control states to both the Driving Command Module and the Vehicle Execution Module. The Vehicle Execution Module, serving as the vehicle’s actuation mechanism, responds to these control states by executing corresponding vehicle actions. This module includes steer-by-wire devices, brake systems, etc., and is tasked with describing the data related to the execution of vehicle actions, such as specifying the vehicle’s turning angle and detailing the response time of the vehicle’s brakes.

**Fig 2:**
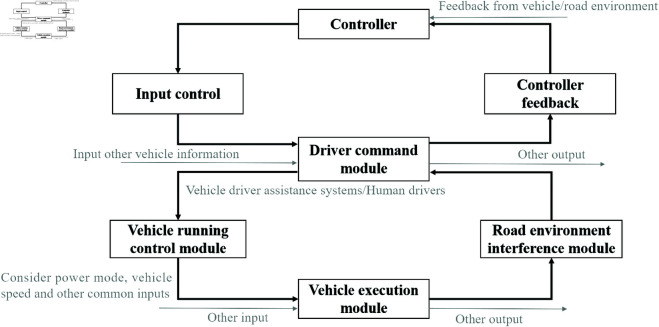
Operation control flow of assisted driving vehicle.

The Road Environment Interference Module is responsible for inputting environmental data that may interfere with the Vehicle Travel Control Module and the Driving Command Module. It is connected to the Vehicle Travel Control Module and provides feedback to the Driving Command Module, as illustrated in Fig 2.

**Fig 3:**
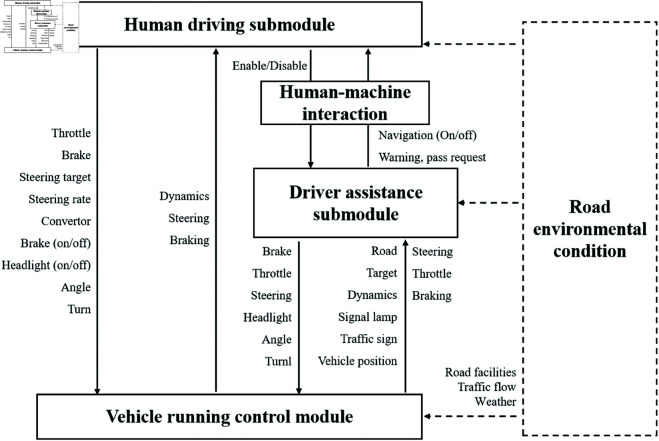
Figure of accident participant categories.

As depicted in Fig 3, the Driving Command Module comprises the Auxiliary Driving Submodule and the Human Driving Submodule. The Human Driving Submodule maintains a direct connection with the Vehicle Drive Control Module. Additionally, it interfaces with the Vehicle Drive Control Module through the Auxiliary Driving Submodule. Furthermore, the Vehicle Drive Control Module is linked to the Vehicle Execution Module. Meanwhile, the Road Environment Interference Module is connected to both the Vehicle Driving Control Module and the Driving Command Module, respectively.

By establishing the logical interconnections among the data modules, the potential operational procedures, control processes, and environmental impact processes of the intelligent driving vehicle prior to the accident are comprehensively encompassed. Prior to conducting the vehicle reliability analysis, a clear depiction of the vehicle’s operational state before the traffic accident is provided, furnishing a solid foundation for the subsequent identification of unsafe factors during vehicle operation.

The Control Action Security Analysis Subsystem is comprised of a blade server equipped with the Control Action Security Analysis tool. A blade server is a server unit capable of accommodating multiple cards within a rack-mounted chassis of standard height, embodying the principles of High Availability and High Density. This server configuration represents a cost-effective platform, particularly suited for intricate traffic accident analysis and high-density computing environments. The blade server boasts an array of data access ports, facilitating access to the Driving Command Module, Vehicle Driving Control Module, Vehicle Execution Module, and Road Environment Interference Module. These ports enable the determination and analysis of unsafe factors contributing to vehicle accidents during the driving process. The blade server analyzes the control actions impacting the safety of the assisted driving vehicle based on the following Control Action Safety Analysis model.

Therefore, in the process of traffic accident investigation of assisted driving vehicles, let *CL*_*i*_be the factors of vehicle assisted driving system involved in the traffic accident,*RL*_*i*_be the factors of human driver, *DL*_*i*_be the factors of road, and *HJ*_*i*_ be the factors of environment, the accident-causing association rule model is built to analyze the comprehensive factors of traffic accident of assisted driving vehicles.


$$f(FD_{i})=\sum_{i\in N}^{k}[\delta_{j}f(CL_{i})+\theta_{j}f(RL_{i})+\rho_{j}f(DL_{i})+\phi_{j}f(HJ_{i})]$$

Let *f*(*FD*_*i*_) be a function of association rule model for the cause of traffic accidents *i*, *M* be collection of factors for assist driving car traffic,*j* is the weight coefficient of causes *i* on traffic accidents of assisted driving vehicles, $\mu_{j}$ is the weight coefficient of function involved vehicle assisted driving system, $\theta_{j}$ is the weight coefficient of function involved human driver, $\rho_{j}$ is the weight coefficient of function involved road, $\phi_{j}$ is the weight coefficient of function involved environment, *f*(*CL*_*i*_) be a function of traffic warning equipment elements variable for *CL*_*i*_, *f*(*RL*_*i*_) be a function of traffic warning equipment elements variable for *RL*_*i*_, *f*(*DL*_*i*_) be a function of traffic warning equipment elements variable for *DL*_*i*_, *f*(*HJ*_*i*_) be a function of traffic warning equipment elements variable for *HJ*_*i*_ .

The feedback mechanism of road environment factors on the operation safety of assisted driving vehicles is analyzed. The road factors involved in assisted driving vehicles are as follows.


$$f(DL_{i})=\sum_{i\in M}[f(JD_{i})+f(DS_{i})+f(RF_{i})+f(DH_{i})+f(BZ_{i})+f(KZ_{i})+f(FB_{i})]$$

Let *f*(*DL*_*i*_) be a function of the road factors involved in assisting the driving of the car, *JD*_*i*_ is static road data interference value,*DS*_*i*_ is the interference value of road attribute data information, *RF*_*i*_ is the interference value of road facility data information, *DH*_*i*_ is the interference value of navigation satellite positioning lane information, *BZ*_*i*_ is the interference value of traffic sign marking information, *KZ*_*i*_ is the traffic signal control facility information interference value, *FB*_*i*_ is the traffic information release facility information interference value.

The environmental factors involved in assisted driving are as follows.


$$f(HJ_{i})=\sum_{i\in M}[f(HV_{i})+f(HT_{i})+f(HR_{i})+f(HW_{i})+f(HN_{i})]$$

Let *f*(*HL*_*i*_) be a function of the road factors involved in assisting the driving of the car, *JD*_*i*_ is static road data interference value,*DS*_*i*_ is the interference value of road attribute data information, *RF*_*i*_ is the interference value of road facility data information, *DH*_*i*_ is the interference value of navigation satellite positioning lane information, *BZ*_*i*_ is the interference value of traffic sign marking information, *KZ*_*i*_ is the traffic signal control facility information interference value, *FB*_*i*_ is the traffic information release facility information interference value.

### 3.3 Reconstruction of accident causal scenarios

The Road Environment Interference Layer Scene Recovery Module of the Traffic Accident Causal Scene Recovery Subsystem utilizes camera devices and graphics simulation software to trace, measure, and analyze the road environment, thereby restoring the traffic accident scene. The Vehicle Control Layer Scene Recovery Module employs onboard electronic data to reconstruct vehicle operational data, encompassing driving processes, collision sequences, collision angles, speeds, acceleration rates, and deceleration rates. The Driving Command Layer Scene Recovery Module, on the other hand, leverages onboard electronic and video data to restore the operational processes of both the auxiliary driving system and the human driver.

The Road Environment Interference Layer Scene Recovery Module achieves a comprehensive restoration of the traffic accident scene through the use of a 360^∘^ automatic camera and 3D digital graphics simulation software. It generates and reconstructs animations, enabling backtracking, measurement, and analysis of the road environment via these animations. The Vehicle Control Layer Scene Recovery Module reconstructs qualitative and quantitative factors such as driving processes, collision sequences, collision angles, speeds, acceleration rates, and deceleration rates, including specific details like wheel turn angles during driving and whether the brake system engages after the driver initiates braking. The Driving Command Layer Scene Recovery Module reconstructs the operational processes of the auxiliary driving system and the human driver through onboard electronic and video data, detailing aspects such as the engagement of the auxiliary driving system, the specific degree of steering wheel rotation by the driver, and the pressure applied to the brake pedal.

**Fig 4:**
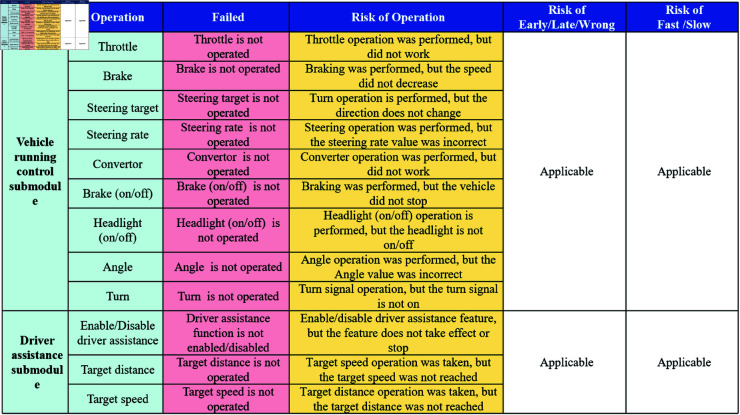
Driving command layer scene recovery module.

**Fig 5:**
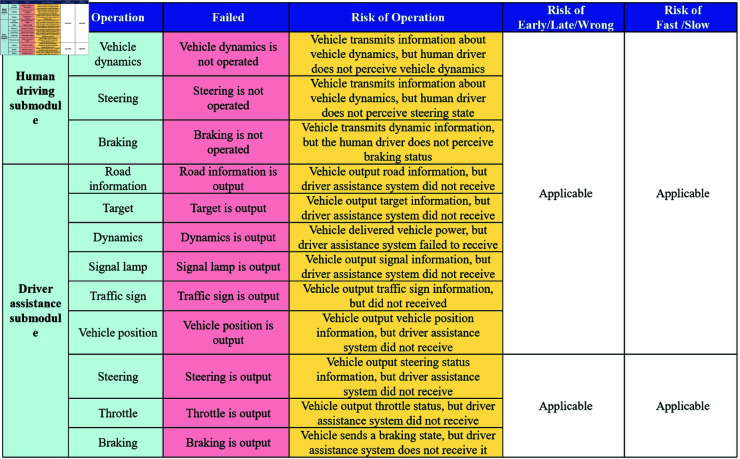
Vehicle control layer scene recovery module.

**Fig 6:**
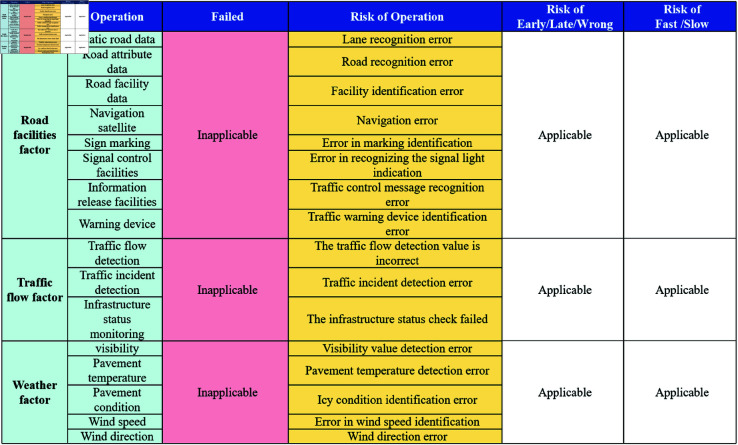
Road environment interference layer scene recovery module.

The Traffic Accident Causal Scene Recovery Subsystem not only restores accident scenes through animations but also describes the working states of individual components, the vehicle’s environment, and the objects within it through textual descriptions. Taking the textual table description as an example, the Driving Command Layer Scene Recovery Module obtains the Driving Command Layer Scene Restoration Content Matrix, as illustrated in Fig 4. Similarly, the Vehicle Control Layer Scene Recovery Module obtains the Vehicle Control Layer Scene Restoration Content Matrix, as shown in Fig 5, and the Road Environment Interference Layer Scene Recovery Module obtains the Road Environment Interference Layer Scene Restoration Content Matrix, as depicted in Fig 6.

### 3.4 Application of results

The investigation report export subsystem comprises the traffic accident in-depth investigation report export module and the traffic accident cause-related list display module. Both modules are interconnected with the traffic accident cause-and-effect scenario recovery subsystem via dedicated data access ports. The embodiment of this invention facilitates the generation of descriptive paragraphs for the reconstructed traffic accident scenes through the traffic accident in-depth investigation report export module. Specifically, these descriptive paragraphs can be formulated as investigation reports based on advanced natural language generation models, such as Chat-GPT, ERNIE Bot, and others. Furthermore, the traffic accident cause-related list display module organizes these paragraph-type investigation reports into a coherent list format.

Utilizing the investigation report export subsystem, an analysis report on the restored traffic accidents can be produced, enabling a direct identification of the core causes, key contributing factors, and the respective degrees of influence of each cause. This streamlined approach significantly aids users and law enforcement in comprehending the underlying origins of traffic accidents. Additionally, the analysis within the report offers reasonable suggestions and serves as a reminder for users and law enforcement to enhance vehicle driving assistance functions and improve road conditions, thereby reducing the likelihood of subsequent traffic accidents and enhancing overall traffic safety.

## 4 Accident case analysis

**Fig 7:**
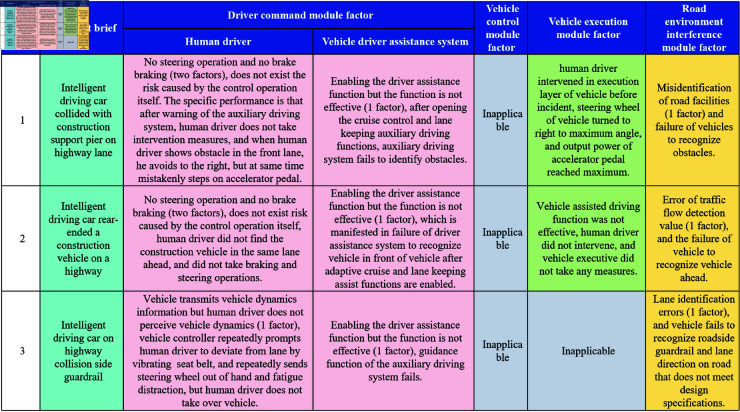
Case analysis of traffic accidents involving assisted driving systems: root cause identification.

The aforementioned method and system were employed to investigate three cases of assisted driving vehicle traffic accidents in China, resulting in the compilation of a comprehensive list detailing the causes of each accident, with each entry representing a distinct traffic incident. A meticulous analysis was conducted to examine the impacts of various factors, including human driver factors, auxiliary driving system factors, vehicle driving control module factors, vehicle execution module factors, and road environment interference module factors, on these traffic accidents. The findings of this analysis are illustrated in Fig 7.

Case 1: Two factors contributed to the human driver’s failure to execute control operations on the vehicle’s driving controller: the omission of a steering maneuver and the neglect of applying the brakes. No intrinsic risk was associated with the control operations themselves. Specifically, despite the assistive driving system issuing a warning, the human driver failed to intervene promptly. Upon noticing an obstacle in the lane ahead, the driver attempted to steer right to avoid it but inadvertently pressed the accelerator pedal simultaneously. Additionally, one factor related to the assistive driving system’s control operations on the vehicle’s driving controller posed a risk: the activation of the assistive driving function without it taking effect. Specifically, after enabling the adaptive cruise control and lane-keeping assist functions, the system failed to recognize the obstacle. Consequently, as the vehicle’s assistive driving function did not operate as intended, the human driver intervened at the execution level prior to the incident, steering the vehicle’s wheel to its maximum angle to the right, with the accelerator pedal output reaching its peak power. Furthermore, one factor pertaining to the road’s static facilities contributed to a risk due to control operations: an error in road facility recognition, which led to the vehicle failing to identify the obstacle.

Case 2: Two factors contributed to the human driver’s failure to execute control operations on the vehicle’s driving controller: the omission of a steering maneuver and the neglect of applying the brakes. No intrinsic risk was associated with the control operations themselves. Specifically, the human driver failed to notice the construction vehicle traveling in the same lane ahead and did not take any braking or steering actions accordingly. Additionally, one factor related to the assistive driving system’s control operations on the vehicle’s driving controller posed a risk: the activation of the assistive driving function without it taking effect. Specifically, after the vehicle had enabled adaptive cruise control and lane-keeping assist functions, the assistive driving system failed to recognize the vehicle ahead. Due to the ineffectiveness of the vehicle’s assistive driving function, the human driver did not intervene, and no measures were implemented at the vehicle’s execution level. Furthermore, one factor pertaining to dynamic traffic information contributed to a risk due to control operations: an error in traffic flow detection values, which led to the vehicle failing to recognize the construction vehicle ahead.

Case 3: The vehicle controller introduced a risk stemming from control operations intrinsic to the human driver, encompassing one primary factor: the vehicle’s transmission of dynamic information that went unperceived by the human driver. Specifically, the vehicle controller repeatedly signaled lane departure through seatbelt vibration and issued numerous alerts for hands-off steering, fatigue, or distraction; however, the human driver failed to assume control of the vehicle. Additionally, the assistive driving system presented a risk related to control operations on the vehicle’s driving controller, including one factor where the assistive driving function was engaged but failed to operate effectively, particularly evidenced by the malfunction of the system’s guidance capability. Furthermore, a risk was associated with control operations inherent to the road’s static infrastructure, involving one factor of incorrect lane recognition, which led the vehicle to overlook roadside barriers and lane orientations that deviated from design specifications on the road in question.

## 5 Discussion and conclusions

The article introduces a Comprehensive Factors Investigation System for Intelligent Connected Vehicle Traffic Accidents (CAIS), and systematically evaluates its advantages in comparison with existing typical systems from multiple dimensions in Table 2. This study develops a tailored comprehensive investigation system specifically for traffic accidents related to intelligent driving, which markedly enhances the accuracy and reliability of investigations concerning assisted driving vehicles. This is achieved through the integration of multi-source heterogeneous data and the application of the Systems-Theoretic Process Analysis (STPA) method for system safety analysis. Grounded in the theoretical innovation of constructing a safety control analysis model, the system comprehensively accounts for diverse influencing factors, including human, vehicle, road, and environmental aspects, in assisted driving vehicle traffic accidents. Furthermore, it innovates in terms of investigation content and methodologies, aligning more closely with the safety requirements for the practical development of intelligent connected vehicles.

**Table 2:** Comparison of existing traffic accident investigation systems with CAIS.

System Type	Main Features	Advantages	Limitations	Comparison with CAIS
Traditional System	Manual inspection, witness statements, physical evidence (skid marks, collision angles)	Mature methodology, universally applicable	Unable to analyze assisted driving systems, subjective judgment reliance	CAIS uses data-driven analysis for precise system failure identification
Data-Driven System	Vehicle EDR/sensor data for quantitative analysis	Objective data, differentiates human/system failures	Data dependency, incomplete/open data issues	CAIS extends data boundaries with logical relationship analysis
Simulation System	Virtual accident scenario replication	Repeatable testing, cost-effective	Accuracy dependent on models/input data	CAIS combines real data with logical relationships for accuracy
Expert System	Multi-disciplinary team evaluation	Comprehensive analysis, legal/insurance applications	Subjective evaluation	CAIS supports expert decisions with objective data frameworks
AI-Assisted System	Machine learning for failure pattern recognition	Efficient data processing, real-time monitoring	Data quality dependency, interpretability issues	CAIS provides interpretable analysis via control action model

In terms of methodological innovation, a holistic investigation process encompassing “evidence extraction, cause analysis, hazard tracing, and responsibility determination" is introduced, addressing the deficiencies of traditional methods in dissecting the multi-factorial correlations in accidents involving assisted driving vehicles. Through the synergistic operation of the end-cloud system, cross-dimensional evidence integration is achieved, spanning from local vehicle data to the broader traffic environment, thereby furnishing high-confidence data support for accident reconstruction.

Regarding technological advancements, the specially designed accident evidence extraction algorithm and multi-factor coupling analysis model adeptly tackle the challenge of causal chain disruption in intricate scenarios. Case studies illustrate that this framework enhances the accuracy of identifying mixed causes, such as human error, sensor malfunction, and road design flaws, when compared to traditional methods, particularly demonstrating robust performance in human-machine hybrid driving contexts.

In respect to application value, the research findings furnish a scientific rationale for determining traffic accident responsibility, aiding judicial decisions, insurance claims, and the optimization of vehicle safety standards. Additionally, the accident database, grounded in the mining of all-factor association rules, establishes a robust data foundation for the iterative upgrading of intelligent driving systems and the formulation of traffic management policies.

The limitations of this study are mainly manifested in two aspects: firstly, the current system training relies on data from specific regional traffic scenarios, and its generalization performance on emerging road infrastructure needs further verification; secondly, although an ethical framework has been introduced to delineate the responsibilities in human-machine co-driving, differences in regulations regarding algorithm transparency and privacy protection across different judicial jurisdictions may affect the model’s universality. Looking ahead, future work will focus on the following directions: constructing a dynamic knowledge graph of accident causes to provide proactive safety warnings for autonomous driving systems; exploring seamless integration with traffic regulations and ethical standards; and promoting the improvement of a “technology-law-society" collaborative governance framework.

## Supporting information

S1 Table Minimal dataset for causal factors in assisted driving accidents. (XLS)

## References

[1] García A, Camacho-Torregrosa FJ, Padovani Baez PV. Examining the effect of road horizontal alignment on the speed of semi-automated vehicles. Accid Anal Prev. 2020;146:105732. doi: 10.1016/j.aap.2020.105732 32853991

[2] Moradloo N, Mahdinia I, Khattak AJ. Safety in higher level automated vehicles: investigating edge cases in crashes of vehicles equipped with automated driving systems. Accident Anal Prevent. 2024;203:107607. doi: 10.1016/j.aap.2024.107607 38723333

[3] Guo Y, Liu P, Yuan Q. Review on research of rond traffic safety of connected and automated vehicles. J Traffic Transp Eng. 2023;23(5):19–38. doi: 10.19818/j.cnki.1671-1637.2023.05.003

[4] Wang XS, Qin DM, Ye XC. Recent developments on road readiness for automated driving. China J Highw Transp. 2024;37(1):175–93. doi: 10.19721/j.cnki.1001-7372.2024.01.015

[5] Chen JQ, Shu XX, Lan FC. Construction of autonomous vehicles test scenarios with typical dangerous accident characteristics. J South China Univ Technol (Nat Sci Ed). 2021;49(5):1–8.

[6] Bhuiyan H, Governatori G, Bond A, Rakotonirainy A. Traffic rules compliance checking of automated vehicle maneuvers. Artif Intell Law. 2023;32(1):1–56. doi: 10.1007/s10506-022-09340-9

[7] Atak A, Kingma S. Safety culture in an aircraft maintenance organisation: a view from the inside. Safety Science. 2011;49(2):268–78. doi: 10.1016/j.ssci.2010.08.007

[8] Leveson NG. Engineering a safer world: systems thinking applied to safety. Cambridge, MA: MIT Press. 2016.

[9] Delikhoon M, Zarei E, Banda OV, Faridan M, Habibi E. Systems thinking accident analysis models: a systematic review for sustainable safety management. Sustainability. 2022;14(10):5869. doi: 10.3390/su14105869

[10] Sha Y, Hu J, Zhang Q, Wang C. Systematic analysis of the contributory factors related to major coach and bus accidents in China. Sustainability. 2022;14(22):15354. doi: 10.3390/su142215354

[11] Hamim OF, Ukkusuri SV. Determining prominent factors across system hierarchies to improve road safety in LMICs: a case study of Bangladesh. Safety Science. 2022;150:105709. doi: 10.1016/j.ssci.2022.105709

[12] Stanton NA, Box E, Butler M, Dale M, Tomlinson E-M, Stanton M. Using actor maps and AcciMaps for road safety investigations: development of taxonomies and meta-analyses. Safety Sci. 2023;158:105975. doi: 10.1016/j.ssci.2022.105975

[13] Wang Q, Mei Q, Liu S, Zhou Q, Zhang J. Demographic differences in safety proactivity behaviors and safety management in Chinese small–scale enterprises. Safety Sci. 2019;120:179–84. doi: 10.1016/j.ssci.2019.06.016

[14] Gonzalez Barman K. Accident causation models: the good the bad and the ugly. Eng Stud. 2023;15(2):75–100. doi: 10.1080/19378629.2023.2230841

[15] Kim H, Han H, You Y, Cho M-J, Hong J, Song T-J. A comprehensive traffic accident investigation system for identifying causes of the accident involving events with autonomous vehicle. J Adv Transport. 2024;2024(1):9966310. doi: 10.1155/2024/9966310

[16] Chubukov A, Kapitanov V, Monina O, Silyanov V, Brannolte U. Calculation of traffic capacity of signaled intersections. Transport Res Procedia. 2017;20:125–31. doi: 10.1016/j.trpro.2017.01.032

[17] Hu L, Bao X, Wu H, Wu W. A study on correlation of traffic accident tendency with driver characters using in-depth traffic accident data. J Adv Transport. 2020;2020:1–7. doi: 10.1155/2020/9084245

[18] Lu J, Peng Z, Yang S, Ma Y, Wang R, Pang Z, et al. A review of sensory interactions between autonomous vehicles and drivers. J Syst Architect. 2023;141:102932. doi: 10.1016/j.sysarc.2023.102932

[19] Tengilimoglu O, Carsten O, Wadud Z. Infrastructure requirements for the safe operation of automated vehicles: opinions from experts and stakeholders. Transport Policy. 2023;133:209–22. doi: 10.1016/j.tranpol.2023.02.001

[20] Nascimento AM, Vismari LF, Molina CBST, Cugnasca PS, Camargo JB, Almeida JR de, et al. A systematic literature review about the impact of artificial intelligence on autonomous vehicle safety. IEEE Trans Intell Transp Syst. 2020;21(12):4928–46. doi: 10.1109/tits.2019.2949915

[21] Chen X, Tang Z, Johansson KH, Mårtensson J. Safe platooning control of connected and autonomous vehicles on curved multi-lane roads. arXiv preprint 2025. https://arxiv.org/abs/2502.10180

[22] Muzahid AJM, Kamarulzaman SF, Rahman MA, Murad SA, Kamal MAS, Alenezi AH. Multiple vehicle cooperation and collision avoidance in automated vehicles: survey and an AI-enabled conceptual framework. Sci Rep. 2023;13(1). doi: 10.1038/s41598-022-27026-9 PMC9837199 36635336

[23] Adnan Yusuf S, Khan A, Souissi R. Vehicle-to-everything (V2X) in the autonomous vehicles domain – a technical review of communication, sensor, and AI technologies for road user safety. Transport Res Interdiscip Perspect. 2024;23:100980. doi: 10.1016/j.trip.2023.100980

[24] Zhu J, Ma Y, Lou Y. Multi-vehicle interaction safety of connected automated vehicles in merging area: a real-time risk assessment approach. Accid Anal Prev. 2022;166:106546. doi: 10.1016/j.aap.2021.106546 34965492

[25] Zhu J, Ma Y, Zhang Y, Zhang Y, Lv C. Takeover quality prediction based on driver physiological state of different cognitive tasks in conditionally automated driving. Adv Eng Inform. 2023;57:102100. doi: 10.1016/j.aei.2023.102100

[26] Ma F, Wang X, Yang W. Real-time accident risk identification for freeway weaving segments based on video analytics. Measurement. 2025;242:115783. doi: 10.1016/j.measurement.2024.115783

[27] Rehman MA, Numan M, Tahir H, Rahman U, Khan MW, Iftikhar MZ. A comprehensive overview of vehicle to everything (V2X) technology for sustainable EV adoption. J Energy Storage. 2023;74:109304. doi: 10.1016/j.est.2023.109304

[28] Zhen L, Wu J, Chen F, Wang S. Traffic emergency vehicle deployment and dispatch under uncertainty. Transport Res Part E: Logist Transport Rev. 2024;183:103449. doi: 10.1016/j.tre.2024.103449

[29] Wang C, Abdel-Aty M, Han L. Tunnel crash severity and congestion duration joint evaluation based on cross-stitch networks. Accid Anal Prevent. 2025;213:107942. doi: 10.1016/j.aap.2025.107942 39908740

[30] Duan XH, Wu JX, Xiong YL. Dynamic emergency vehicle path planning and traffic evacuation based on salp swarm algorithm. J Adv Transp. 2022;2022:1–28. doi: 10.1155/2022/7862746

[31] Li H, Bin Kaleem M, Liu Z, Wu Y, Liu W, Huang Z. IoB: internet-of-batteries for electric vehicles–architectures, opportunities, and challenges. Green Energy Intell Transp. 2023;2(6):100128. doi: 10.1016/j.geits.2023.100128

[32] Miller T, Durlik I, Kostecka E, Borkowski P, Łobodzińska A. A critical AI view on autonomous vehicle navigation: the growing danger. Electronics. 2024;13(18):3660. doi: 10.3390/electronics13183660

